# Assessment of Delivery Quality Assurance for Stereotactic Radiosurgery With Cyberknife

**DOI:** 10.3389/fonc.2021.751922

**Published:** 2021-11-17

**Authors:** Jun Li, Xile Zhang, Yuxi Pan, Hongqing Zhuang, Junjie Wang, Ruijie Yang

**Affiliations:** Department of Radiation Oncology, Peking University Third Hospital, Beijing, China

**Keywords:** delivery quality assurance, Cyberknife, small field, ionization chamber, EBT3 film

## Abstract

**Purpose:**

The purpose of this study is to establish and assess a practical delivery quality assurance method for stereotactic radiosurgery with Cyberknife by analyzing the geometric and dosimetric accuracies obtained using a PTW31016 PinPoint ionization chamber and EBT3 films. Moreover, this study also explores the relationship between the parameters of plan complexity, target volume, and deliverability parameters and provides a valuable reference for improving plan optimization and validation.

**Methods:**

One hundred fifty cases of delivery quality assurance plans were performed on Cyberknife to assess point dose and planar dose distribution, respectively, using a PTW31016 PinPoint ionization chamber and Gafchromic EBT3 films. The measured chamber doses were compared with the planned mean doses in the sensitive volume of the chamber, and the measured planar doses were compared with the calculated dose distribution using gamma index analysis. The gamma passing rates were evaluated using the criteria of 3%/1 mm and 2%/2 mm. The statistical significance of the correlations between the complexity metrics, target volume, and the gamma passing rate were analyzed using Spearman’s rank correlation coefficient.

**Results:**

For point dose comparison, the averaged dose differences (± standard deviations) were 1.6 ± 0.73% for all the cases. For planar dose distribution, the mean gamma passing rate for 3%/1 mm, and 2%/2 mm evaluation criteria were 94.26% ± 1.89%, and 93.86% ± 2.16%, respectively. The gamma passing rates were higher than 90% for all the delivery quality assurance plans with the criteria of 3%/1 mm and 2%/2 mm. The difference in point dose was lowly correlated with volume of PTV, number of beams, and treatment time for 150 DQA plans, and highly correlated with volume of PTV for 18 DQA plans of small target. DQA gamma passing rate (2%/2 mm) was a moderate significant correlation for the number of nodes, number of beams and treatment time, and a low correlation with MU.

**Conclusion:**

PTW31016 PinPoint ionization chamber and EBT3 film can be used for routine Cyberknife delivery quality assurance. The point dose difference should be within 3%. The gamma passing rate should be higher than 90% for the criteria of 3%/1 mm and 2%/2 mm. In addition, the plan complexity and PTV volume were found to have some influence on the plan deliverability.

## Introduction

Stereotactic radiosurgery (SRS) and stereotactic body radiotherapy (SBRT), with delivery of high biological effective dose (BED) and enhanced antitumor effect to improve the rate of local control or pain relief (1), have emerged as effective adjuvant and standalone treatment options for many kinds of cancers. To date, it has demonstrated a significant advantage over conventional external beam radiation therapy for relatively radioresistant tumors, such as non-small cell lung cancer and renal cell carcinoma (2, 3). However, high profits also face with high risks. The planning and treatment process for SRS and SBRT is complex, and the potential for serious toxicity uncommonly observed in conventional radiotherapy has been observed (4). A focus on treatment quality and safety is needed to achieve the favorable rates of local control and less toxicity. Therefore, SRS and SBRT demand high geometric and dosimetric accuracy for treatment delivery. Especially for high fraction dose radiotherapy with complex systems such as the Cyberknife (Accuray Incorporated, Sunnyvale CA, USA), a practical and efficient method for routine pretreatment verification is needed (5).

Cyberknife system can produce very sharp fall-off dose distribution with many small and non-isocentric non-coplanar beams (6). As field size decreases further, the response of the detector changes more rapidly, and the effects of measurement uncertainties become increasingly significant. The use of small beams in radiation therapy techniques has increased substantially in Cyberknife, particularly for SRS and SBRT. Delivery quality assurance (DQA)for conventional linear accelerators with multi-leaf collimators (MLC) is performed using two- (2D) or three-dimensional (3D) ionization chamber or diode arrays. However, the spacing and spatial resolution of diode or ionization chamber arrays are routinely too large for the small beams of Cyberknife (7). Although a previous study has provided test scenarios for Cyberknife DQA with high resolution liquid-filled ionization chamber array, the results indicated that different incident angles and source-axis distance (SAD) variations influenced its dose response (7). There were no angular correction factors to overcome the beam orientation susceptibility for this DQA technique currently, especially for Cyberknife with non-isocentric non-coplanar beam arrangements (8). Besides, ArcCHECK cylindrical 3D-array was also tested for the Cyberknife pretreatment DQA. The angular dependence of 3% was observed for angle up to 6° when irradiated with FFF beam (9). Furthermore, the non-water equivalency of the diode engenders over-response for small field dose measurement. The small and non-isocentric non-coplanar beams of Cyberknife are characterized by high-dose gradient and a lack of lateral charged particle equilibrium. Therefore, a dosimetric tool with high spatial resolution, tissue equivalence, and directional independence is required (10).

For Cyberknife radiosurgery, the American Association of Physicists in Medicine (AAPM) task group (TG) 135 report recommends performing DQA using radiochromic films (11). EBT3 film has excellent spatial resolution and tissue equivalence. Furthermore, it has the characteristics independent of beam angle, energy, and dose rate (12). Some studies have tested EBT3 films in the verification of high doses delivered to lesions with complex shapes for Cyberknife (6, 13). Several factors of uncertainties associated with the film for dosimetric measurement, such as scanner, background, and film uniformity, impact the dosimetric accuracy. The film is commonly used for relative dose analysis.

AAPM TG 135 recommended conducting a DQA test for patients intending to undergo CyberKnife SRS or SBRT in order to comprehend the overall accuracy of dose delivery. The acceptance criteria of more than 90% gamma passing rate at 2%/2 mm for the tumor and critical structures were recommended (11). Cyberknife system uses non-isocentric multidirectional small size beams and fluctuating beam intensities to realize complex dose distribution (14). The overall dose accuracy should be verified similarly to that of intensity-modulated radiation therapy (IMRT), including the absolute point dose in target volume. However, some studies implemented DQA just to analyze the planar dose distribution using EBT films, without independent absolute point dose verification (15).

Complexity metrics related to the dosimetry accuracy are valuable tools for improving plan optimization and validation and providing guidance when there is a conflict between plan quality (regarding the achievement of plan goals) and plan complexity (16, 17). For Cyberknife based on multileaf collimator (MLC), approaches of estimating plan complexity have been studied (18). However, there are no studies for evaluating the complexity of CK based on IRIS treatment plans.

The aim of this study is to evaluate two different DQA systems (PinPoint ionization chamber and BET3 films) for measuring point dose and planar dose distribution simultaneously for the Cyberknife pretreatment verification in various treatment sites, to establish a practical and efficient DQA method for Cyberknife system. Moreover, our study explores the relationship between parameters of plan complexity, target volume, and deliverability parameters of DQA to provide a valuable reference for improving plan optimization and validation.

## Methods and Materials

### Cyberknife System

The VSI Cyberknife system (Accuray Inc., CA, USA) has a compact 6 MV linear accelerator (1,000 MU/min LINAC), mounted on a six-axis robot controlled by a computer. Secondary collimators including fixed and IRIS collimators feature 12 various aperture sizes (diameter range from 5 to 60 mm at a distance of 800 mm from the source). The treatment couch can move in three translational and two rotational directions. Cyberknife contains a high-resolution image-guided tracking system, which consists of a pair of orthogonally positioned detectors and X-ray sources. Image-guided tracking system can acquire a pair of live images during treatment at given time intervals. The live images are registered with DRR images generated from the planning CT data set. The online-detected offsets from the patient’s planning position are used to track the target automatically.

### Patients, Treatment Plans, Targets, and Collimators Used

One hundred fifty patients undergoing SRS/SBRT with Cyberknife between December 2017 and January 2019 in our institute were included in this study. Treatment plans implemented aim at both radical and palliative care. The treatment sites included 64 cases of the spine, 37 cases of the lung, 28 cases of the brain, and 21 cases of the abdomen (including liver, pancreas, and retroperitoneal). The target volume (TV) ranged from 0.73 to 273.2cc for all the tumors. The target volume, prescription dose, and aperture of collimators for all the DQAs plans of different sites are shown in **Table 1**.

**Table 1:** The target volume and aperture of collimators for all the DQA plans of different sites.

Site of tumor	Spine	Brain	Lung	Abdomen
Target volume (cc) (median [range])	73.53 [14.9-118.39]	28.06 [0.73-68.97]	87.74 [21.72-243.13]	79.01 [63.02-273.2]
Aperture of collimators (mm) (median [range])	25 [10-50]	20 [10-35]	25 [12.5-50]	30 [12.5-50]
Treatment time of per fraction (min)	41 [29-51]	33 [24-47]	42 [27-53]	39 [33-55]
Prescription dose (Gy)	27 [19-40]	21 [18-35]	30 [19-60]	35 [24-48]
Number of fractions	3 [1-5]	3 [1-5]	3 [1-7]	3 [1-6]
Isodose line	76 [68-85]	70 [61-78]	78 [71-88]	75 [70-79]

### Delivery Quality Assurance

A DQA plan was performed by exposing a Gafchromic EBT3 film (Ashland Incorporated, NJ, USA), and simultaneously irradiating a PTW31016 PinPoint ionization chamber (sensitive volume, 0.016 cm^3^; length of sensitive volume, 2.9 mm; PTW Inc., Freiburg, Germany) inserted into a stack of RW3 solid phantom (thickness, 1cm; PTW Inc., Freiburg, Germany) including four fiducial markers for beam delivery tracking. The expected dose to the film and ionization chamber were calculated using treatment planning system (TPS) by overlaying the treatment plan onto the CT images of the solid phantom. The objective of DQA was to check the accuracy of dose calculation and delivery for the pretreatment plan.

### Layout of Phantom and Detectors

**Figure 1:**
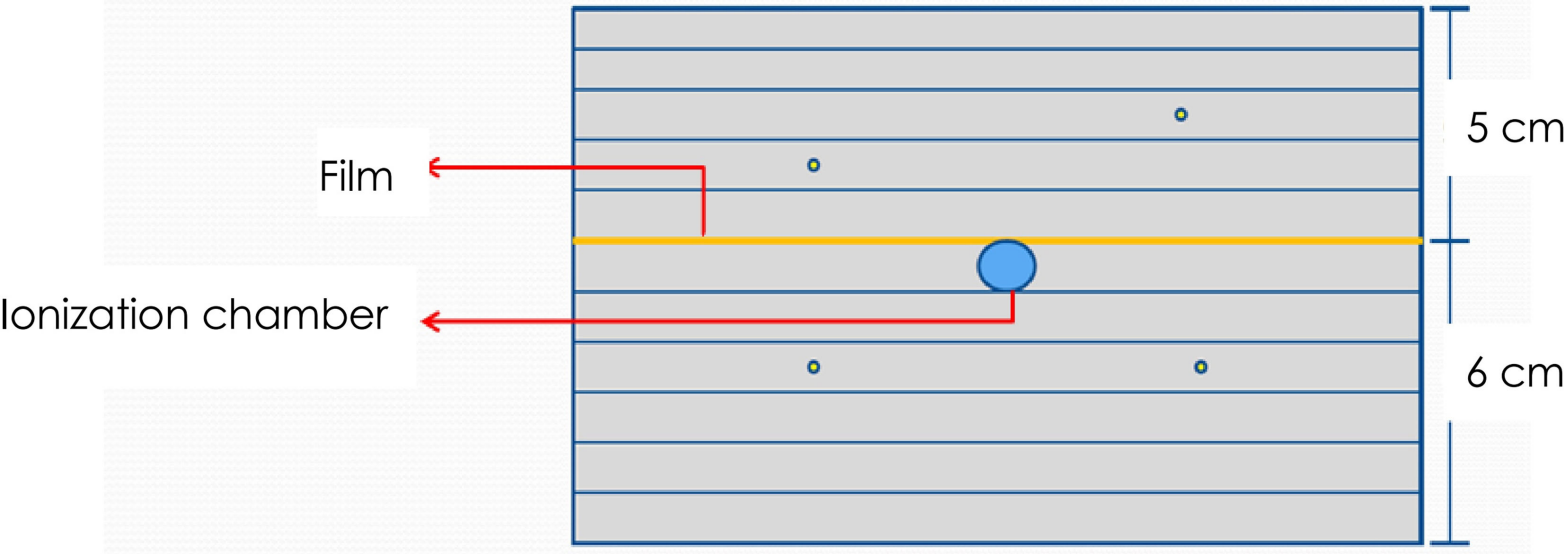
Schematic layout of the dose measurement using the PinPoint ionization chamber and EBT3 films. A total of 4 fiducial markers were implanted into the phantom to facilitate automatic phantom set-up by fiducial tracking.

A stack of RW3 phantom and a PTW31016 PinPoint ionization chamber was used for absolute point dose verification and EBT3 films for planar dose distribution verification. Four fiducial markers were implanted into the phantom for automatic fiducial tracking. A schematic layout of phantom, the ionization chamber, and film is shown in **Figure 1**.

The phantom is a solid water phantom of 30 cm × 30 cm × 11 cm. A hole was drilled in the center of the phantom to allow for the insertion of a PinPoint ionization chamber for point dose measurement. This hole was shaped to fit the chamber. The top of the ionization chamber was tightly attached to the hole to ensure the repeatability and stability of the ionization chamber position. The phantom is divided into two halves so that a sheet of film can be put on the central plane of the phantom at the depth of 5 cm.

### DQA Plans

One hundred fifty pretreatment DQAs were performed for each clinical plan. The selected plans had been optimized for different treatment sites using either fixed collimators or IRIS collimators. The delivery time of the DQA plans ranged from 18 to 53 min. The fractional dose of DQA plans ranged from 5 to 21 Gy. By rescale operation, the fractional dose was reduced to <10 Gy.

For DQAs, the absolute point dose was measured at the high-dose region of the target volume (TV). The fiducial tracking DQA plans were created in three steps. First, the CT images of the pretreatment plan were registered with the CT images of RW3 solid phantom. There is a distance between the ionization chamber center of the RW3 solid phantom and the imaging center of the treatment locating system. Thus, the PTV center of pretreatment planning images was moved to the ionization chamber center of RW3 phantom planning images. The ionization chamber center was located in homogeneous dose region (ideally <3%). Second, information of beams such as robotic manipulator node coordinates, target coordinates, collimator size, and monitor units were identically transferred to CT images of the RW3 solid phantom. Third, the dose distributions were recalculated on the phantom. Finally, the measured point dose was compared with the calculated mean dose at the sensitive volume of the ionization chamber.

Evaluation was limited when high-dose regions of small targets were not on film plane. Thus, after the DQA plan was generated, it was necessary to check the dose distribution to ensure that the high-dose region of the target was on the film plane. If the high-dose region was not on film plane, we would shift the target center to the film plane to generate another DQA plan for planar dose measurements.

### Film Calibration and Scanning

The EBT3 film signal-to-dose conversion and planar dose distribution analysis was performed with RIT113 software (Radiological Imaging Technology, Inc., USA, version 11.0). Dose calibration of the EBT3 film was performed using a stack of RW3 phantom. A calibration curve was generated by exposing 16 film pieces at different dose levels between 0 and 10 Gy. The calibration films were irradiated under the vertical beam with fixed collimator aperture of 60 mm at the depth of 1.5 cm. Films were scanned in red–blue–green (RGB) format on a flatbed color scanner to build calibration curve relating the absorbed dose to the response of the film in each of the color channels. The absorbed dose delivered to each film was measured using the PTW30013 ionization chamber. The 16 films were scanned using the Expression 10000XL scanner (Epson America Inc., Long Beach, CA, USA) 24 h after irradiation. The short edge of the film was positioned paralleling to the scan direction to avoid lateral scanning effects. The color scanning was performed at 48 bits and a spatial resolution of 72 dpi without color correction, and all images were saved in TIFF format. It is very important to incorporate the impact of time-dependent change on optical density for dose analysis using EBT3 film (13). To avoid failure of film analysis due to different development times of calibration and patient film, wait 24 h after plan irradiation to achieve saturation before scanning the patient film. The median time of plan irradiation and patient film analysis is 27.3 h, range from 25.5 to 31.7 h.

### Point Dose Measurement

For absolute point dose measurement, a PTW31016 PinPoint ionization chamber was used. The measured point dose was compared with the calculated mean dose at the sensitive volume of the PinPoint ionization chamber by a MultiPlan 4.6 (Accuray Inc., CA, USA) TPS. The percent dose difference ratio between ion chamber measured and calculated was defined as follows:


$$\text{Dose difference}=\frac{\text{Dos}{\text{e}}_{\text{cal}}-\text{Dos}{\text{e}}_{\text{meas}}}{\text{Dos}{\text{e}}_{\text{cal}}}\times 100$$

where is the measured point dose, and is the calculated dose at the same position as the point of measurement for. The results were considered to have been passed when the dose difference was <3%.

### Gamma Index Analysis

To quantify the differences between the calculated and measured dose distributions, the gamma index analysis was performed to determine the agreement. Gamma index analysis was performed using RIT 113 software (RIT, Inc. Co, USA).

AAPM TG 135 recommends the 2%/2 mm gamma index criteria and requires the gamma passing rate above 90%. The criteria of 2 mm was appropriate due to the intrinsic uncertainties of the analysis in addition to the system delivery inaccuracy for film dosimetry. The stricter DTA criterion of 1 mm was also used considering of the Cyberknife nominal accuracy of submillimeter (6). The passing rate *via* gamma analysis was calculated with gamma value <1 (γ < 1). The results were considered to have been passed when the passing rate was above 90% for the criteria of 3%/1 mm and 2%/2 mm. Before gamma index analysis, film dose map and the 2D calculated dose map were aligned by auto-alignment function of RIT 113 software. Among the registration methods provided by RIT 113, we use the maximum dose registration method. Through calculation, RIT 113 software selected multiple maximum dose points on several regions of the local dose plane between film dose map and 2D calculated dose map, respectively. Based on these maximum dose points of two maps, RIT 113 performed automatic an alignment.

### Complexity Metrics

The software of MATLAB (2010a MathWorks Inc., USA) was used to read and process the information of DICOM-RT files to obtain the complexity metrics, including monitor units (MUs), number of beams, number of nodes, and treatment time.

### Statistical Analysis

The statistical significance of the correlations between the parameters of complexity metrics, target volume, and the gamma passing rate were analyzed using Spearman’s rank correlation coefficient and the threshold of p < 0.05. The low, moderate, and high correlations were, respectively, defined for values of |r| < 0.4, 0.4 ≤ |r| ≤ 0.7, and |r| > 0.7.

## Results

### Point Dose Measurements

**Figure 2:**
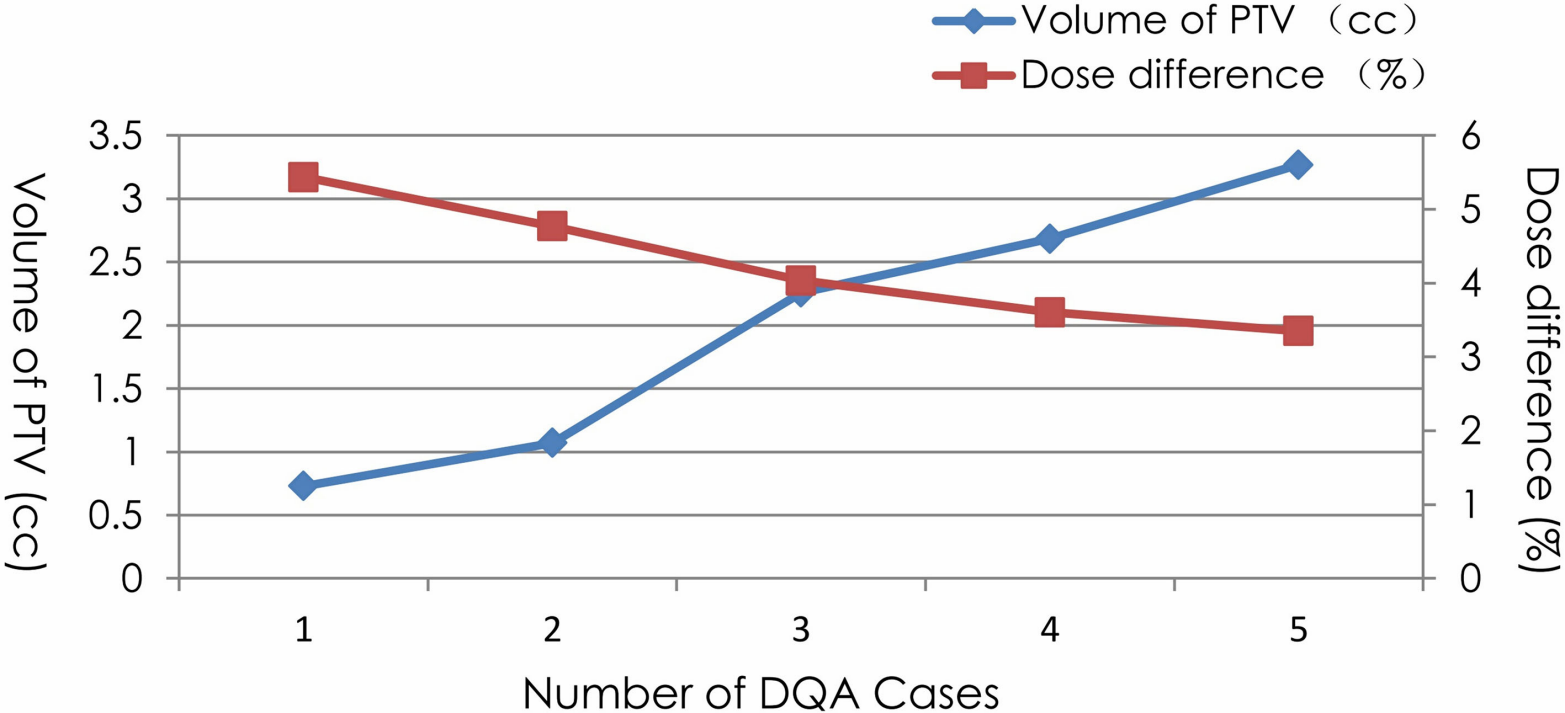
Characteristics of PTV volume and point dose difference for 5 cases dissatisfying with the acceptance criteria.

The point dose difference of the spine, lung, brain, and abdomen DQAs were 1.44% ± 0.76%, 1.71% ± 0.58%, 1.94% ± 1.33%, and 1.57% ± 0.59%, respectively. The dose deviations of brain and lung DQAs were higher than the dose deviations of spine and abdomen DQAs. In addition, there were five cases in which dose differences were more than 3%. Characteristics of target volume and point dose difference for the five cases without achieving the acceptance criteria of point dose were given in **Figure 2**. The large deviation for the point dose measurements was found in the DQAs, in which target volumes were very small. The dose difference increased with the decrease in target volume.

### Planar Dose Measurements

**Figure 3:**
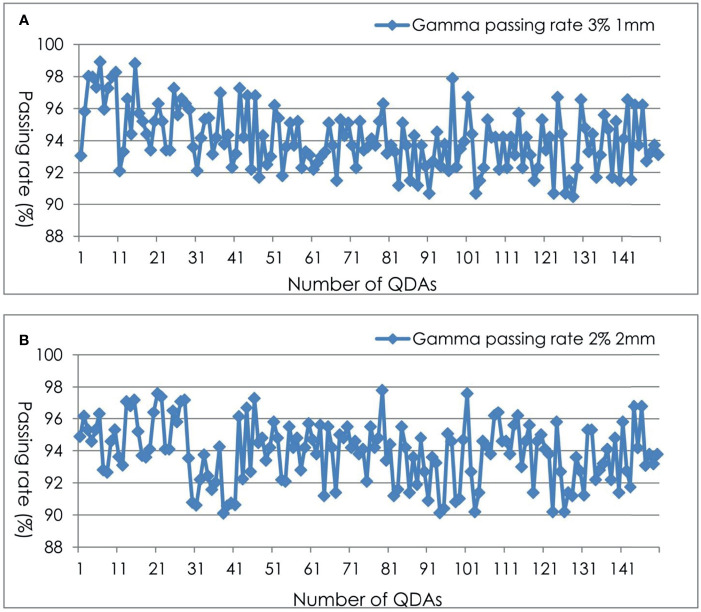
Gamma passing rate for film-based Cyberknife DQAs at 3%/1mm **(A)**, 2%/2 mm **(B)**.

For the 150 planar dose distribution pretreatment DQAs performed with EBT3 films for different treatment sites, a summary of the passing rate *via* gamma index analysis using two criteria is shown in **Table 2** and **Figure 3**. For film-based Cyberknife DQAs at 3%/1 mm, 2%/2 mm, the mean and standard deviation of the passing rate for all the DQAs were 94.26% ± 1.89% and 93.86% ± 2.16% respectively.

**Table 2:** The mean and standard deviation of the passing rate based on the gamma index analysis.

Treatment sites	Spine	Brain	Lung	Brain	Abdomen	Total
3%/1 mm	93.82% ± 1.54%	95.85% ± 1.90%	93.36% ± 1.78%	95.85% ± 1.90%	94.01% ± 1.70%	94.26% ± 1.89%
2%/2 mm	93.44% ± 2.0%	94.51% ± 1.79%	91.60% ± 2.37%	94.51% ± 1.79%	93.58% ± 1.69%	93.86% ± 2.16%

### Correlation Between Plan Complexity Metrics, Target Volume, and Gamma Passing Rate

**Figure 4:**
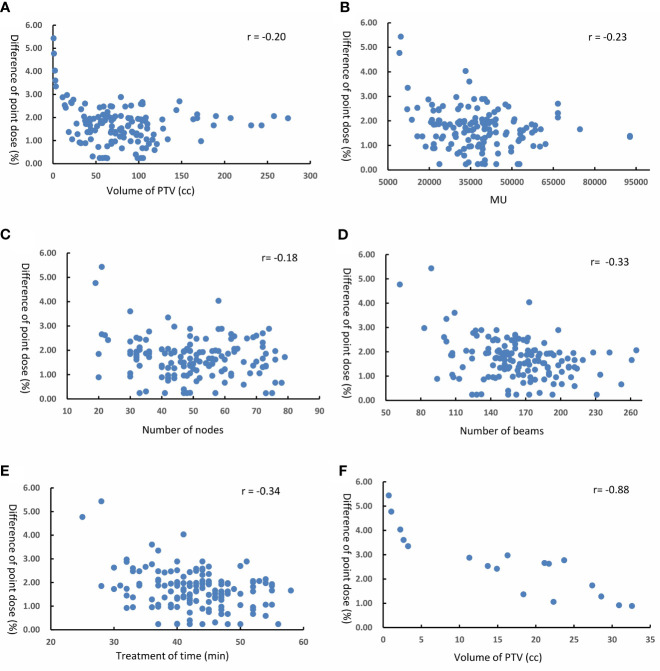
Difference of point dose as function of **(A)** volume of PTV **(B)** MU **(C)** number of nodes **(D)** number of beams **(E)** treatment time in 150 DQA plans, and **(F)** volume of PTV in 18 DQA plans.

**Figure 4** shows the scatter diagram and correlation coefficients for point dose difference as function of volume of PTV, MU, number of nodes, number of beams, and treatment time. We could get that the difference in point dose was lowly correlated with the volume of PTV(r = −0.20, p = 0.048), number of beams (r = −0.33, p = 0.037), and treatment time (r = −0.34, p = 0.041) for 150 DQA plans. There was no correlation between the difference in point dose and MU (r = −0.23, p = 0.073) and the number of nodes (r = −0.18, p = 0.182). For 18 DQA plans of small tumor, when PTV volume was <32.6 cc, the difference in point dose and volume PTV displayed a high correlation (r = −0.88, p = 0.002) and had a negative correlation.

**Figure 5:**
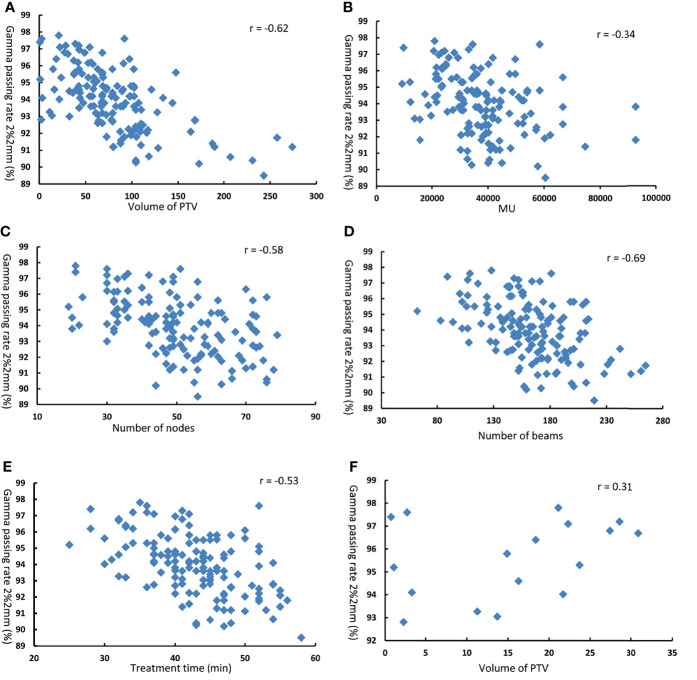
Gamma passing rate at 2% 2mm as function of **(A)** volume of PTV **(B)** MU **(C)** number of nodes **(D)** number of beams **(E)** treatment time in 1 50 DQA plans. and ,**(F)** volume of PTV in 18 DQA plans.

**Figure 5** shows the scatter diagram and correlation coefficients for gamma passing rate at 2%/2 mm criteria as function of volume of PTV, MU, number of nodes, number of beams, and treatment time. The Pearson’s correlation analysis between plan complexity and DQA gamma passing rate (2%/2 mm) showed a moderate significant correlation for number of nodes (r = −0.58, p = 0.032), number of beams (r = −0.69, p = 0.009), and treatment time (r = −0.53, p = 0.012), and a low correlation for MU (r = −0.34, p = 0.046). Meanwhile, there was moderate significant correlation between gamma passing rate (2%/2 mm) and volume of PTV(r = −0.62, p = 0.018). For 18 DQA plans of small tumor, there was no significant correlation between gamma passing rate and PTV volume (r = 0.31, p = 0.082).

## Discussions

Overall, high point dose accuracy and gamma passing rate were obtained for the 150 cases of DQA of Cyberknife using PinPoint ionization chamber and EBT3 films. The accuracy of Cyberknife delivery is influenced by multiple systematic and random error-related uncertainties. The deviations between measured and calculated point dose and planar dose distribution could be caused by the following reasons.

For point dose, the dose deviation was larger for the brain and lung than for the spine and abdomen. Deviation larger than 3% was found for five cases, in which target volumes were very small. The delivery logbook indicated that these DQAs were all related to non-isocentric non-coplanar plans generated for small targets using collimators smaller than 1.5 cm, such as 1.0 or 1.25 cm. As the target was very small, the treatment plan for these cases exclusively used collimators smaller than 2.0 cm. The dose difference increased with the decrease in target volume. This is perhaps due to volume averaging effects combined with the difference in the fluence perturbation caused by the presence of the chamber in the small fields and the reference field for output factor measurement. Previous studies showed that significant differences between the measurement results obtained by PTW31016 ionization chamber and calculation results, particularly for the small field size for which the difference of the measured output factors (OFs) can reach more than 10% (6). Small field dose measurements are difficult mainly due to the high dose gradient and the lack of lateral electronic equilibrium (19). The dosimeters used for such measurements require ideal characteristics such as owning tissue equivalence, having small sensitive volume, and exhibiting dose rate, energy, direction independence, and ability for high spatial resolution measurement (20, 21). Although the sensitive volume of PTW31016 detector is very small, ideally, it is only suitable for field size larger than 2 cm × 2 cm with regard to absolute dose measurement (22, 23). Besides, the ionization chamber is not tissue equivalent. The presence of air within the phantom results in electron transport alterations (24). Consequently, PTW31016 is not the ideal dosimeter for small field measurements, especially when the field size is <2 cm. Accurate dosimetric evaluation was limited or not possible when high-dose gradient regions of small targets were measured using the PTW31016 ionization chamber.

In addition, the Cyberknife system delivers the intended dose *via* non-isocentric dose delivery. For non-isocentric dose delivery, targeting accuracy is a very important parameter with regard to ensuring delivery of the prescribed dose, given the steep peripheral dose fall-off. Targeting accuracy within 0.25 mm and overall targeting accuracy of 0.29 ± 0.10 mm was reported for fiducial tracking with Cyberknife (24, 25). However, even a submillimeter targeting error could significantly affect the accuracy of dose delivery in a non-isocentric treatment plan. An approach of compensating potential error (PE) was suggested in which the worst-case dose deviation scenario was considered by calculating the maximum dose increase and decrease *via* modification of the source to axis distance (SAD) and off axis distance (OAD) for each beam by ±1.0 mm (26).

For planar dose distribution, the results of relatively low passing rate could derive from the uncertainties of film dosimetry. Scanner, background, and film uniformity could impact the dosimetric accuracy (13, 27). Another important factor is scan time dependence of film. Studies have shown that the dose variation was >10% if the film was scanned in the first 2 h after irradiation and within 3% after 24 h (28). Although an extended post-irradiation delay can be helpful in reducing the measurement uncertainty, the actual process of post-exposure polymerization never stops for the EBT3 film model. Thus, the uncertainty could be reduced by matching the post-irradiation delay for film measurement with the post-irradiation delay for film calibration. A single scan method was also suggested to reduce the film dosimetry uncertainty (29).

For the impact of plan complexity parameters and target volume on the difference of point dose, results of correlation analysis demonstrated that the complexity parameters and PTV volumes were low for all DQA plans. The reason may be that the measurement result of point dose is part of beams from DQA plan. However, for DQA plans of small target, there was high negative correlation between difference of point dose and target volume. That is because the plan of small target used more collimators with small aperture. Small fields cause more difficulties and uncertainties in the accuracy of dose measurement. Thus, as a plan with more small fields, more consideration should be given to the choice of collimator size in the design of plan. For the impact of plan complexity parameters and target volume on the gamma passing rate, results of correlation analysis displayed that as the target area increases, the complexity of the plan increases, and the gamma pass rate of DQA will be lower. Many factors affect the complexity of the plan, and these factors may also affect each other. We generally believe that a relatively better plan verification result can be obtained when the complexity is reduced. Under the condition of meeting the clinical requirements, the complexity of the plan can be reduced by adjusting the relevant parameters of the plan, which can reduce a lot of uncertainty.

SRS/SBRT with Cyberknife is complex and involves a lot of hardware and software factors. The delivery errors can occur in many aspects, such as treatment manipulator errors, the daily output drift, and TPS calculation accuracy (30, 31). CyberKnife delivery accuracy relies on the orthogonal kilovoltage X-ray imaging system and the robotic and beam line accuracy. The imperfect calibration and maintenance of the kV X-ray imaging system and the robotic system can lead to a systematic error in the DQA. In this study, the IRIS cones were used in most treatments, which is a variable aperture collimator using two sets (upper and lower banks) of six tungsten segments to create 12-sided variable sizes field. This IRIS changes the field size during the treatment. For treatment manipulator systems, if IRIS positioning accuracy changed, it would influence the dose distribution. The dose calculation accuracy of TPS depends on the beam model and dose calculation algorithms. For the Multiplan TPS, there were two dose calculation algorithms, namely, the Ray–Tracing (RT) and the Monte-Carlo (MC). The Monte-Carlo algorithm was proved to be more accurate, especially for heterogeneous media (32). For insuring the accuracy of Multiplan TPS, beam data acquisition, modeling, validation, commissioning, and establishment of baseline routine QA datasets were implemented following the related guidelines and recommendations (33–35).

We proposed and evaluated a method with regard to simultaneous measurement for both point and planar dose distribution. Great care and cautions were advised on film calibration and scan, selection and use of ion chamber, and results evaluation. This proposed method for Cyberknife delivery quality assurance was validated in a considerable number and various types of clinical plans. The suggested measurement tools and quality references could be used as a practical and efficient protocol for Cyberknife delivery quality assurance. In our center, we compute the gamma with a dose threshold of 10% due to uncertainty in the low dose region for film dosimetry, considering the clinical significance. The tolerance level was a gamma passing rate above 90% at 3%/1 mm for gamma index analysis. The stricter DTA criterion (1 mm) for DQA validation reflected the Cyberknife nominal submillimeter accuracy more appropriately than the suggested 2 mm. The local percent dose difference (LPDD) criterion (3%), on the other hand, was chosen, as this is generally accepted in absolute film dosimetry due to intrinsic uncertainties of the analysis (15, 16) and system delivery inaccuracy, which is the main reason for performing a DQA analysis. The 3%/1 mm criteria reflected the actual uncertainty of film-based DQA and Cyberknife beam delivery more realistically. This tolerance level was validated on a large number of DQA. Therefore, besides AAPM TG135 recommended 2%/2mm criteria, we suggest that gamma passing rates above 90% using 3%/1 mm criteria can be also used for Cyberknife DQA.

## Conclusion

PTW31016 PinPoint ionization chamber and EBT3 film can be used for routine Cyberknife DQA. The point dose difference should be within 3%. The gamma passing rate should be >90% for the criteria of 3%/1 mm and 2%/2 mm. In addition, the plan complexity and PTV volume were found to have some influence on the plan deliverability. The presented correlations identified between various parameters could be utilized to enhance the efficiency of the radiotherapy process from CK treatment planning to patient DQA.

## Data Availability Statement

The original contributions presented in the study are included in the article/supplementary material. Further inquiries can be directed to the corresponding author.

## Author Contributions

JL conceived and designed the study. JL, XZ, and YP performed the experiments. JL wrote the paper. RY, JW, and HZ reviewed and edited the manuscript. All authors contributed to the article and approved the submitted version.

## Funding

This work was supported by the National Key Research and Development Program (No. 2020YFE0202500), Beijing Municipal Commission of Science and Technology Collaborative Innovation Project (Z201100005620012), Capital’s Funds for Health Improvement and Research (2020-2Z-40919), and China International Medical Foundation (HDRS2020030206).

## Conflict of Interest

The authors declare that the research was conducted in the absence of any commercial or financial relationships that could be construed as a potential conflict of interest.

## Publisher’s Note

All claims expressed in this article are solely those of the authors and do not necessarily represent those of their affiliated organizations, or those of the publisher, the editors and the reviewers. Any product that may be evaluated in this article, or claim that may be made by its manufacturer, is not guaranteed or endorsed by the publisher.
